# Caregivers’ burden and fatigue during and after patients’ treatment with concomitant chemoradiotherapy for locally advanced head and neck cancer: a prospective, observational pilot study

**DOI:** 10.1007/s00520-019-04700-9

**Published:** 2019-02-22

**Authors:** Simone M. C. H. Langenberg, Carla M. L. van Herpen, Claudia C. M. van Opstal, Anke N. M. Wymenga, Winette T. A. van der Graaf, Judith B. Prins

**Affiliations:** 1 Department of Medical Oncology, Radboud University Medical Center, Nijmegen, The Netherlands; 2 Department of Medical Oncology, Medisch Spectrum Twente, Enschede, The Netherlands; 3 Department of Medical Psychology, Radboud University Medical Center, P.O. Box 9101, 6500 HB Nijmegen (840), The Netherlands

**Keywords:** Caregiver, Chemoradiotherapy, LAHNC, Fatigue, Burden

## Abstract

**Purpose:**

Knowledge of caregivers’ burden and fatigue before and after patients’ treatment for locally advanced head and neck cancer is scarce. Therefore, we aimed to explore caregivers’ fatigue and burden in relation to patients’ fatigue, distress, and quality of life.

**Methods:**

For caregivers, burden and fatigue were assessed. For patients, fatigue severity, distress, and health-related quality of life (HRQoL) were assessed. Measurements were conducted prior to treatment, 1 week, and 3 months after chemoradiotherapy.

**Results:**

Caregivers’ burden and fatigue followed patients’ high peak in distress, fatigue, and diminished HRQoL as a consequence of treatment. Caregivers’ baseline fatigue was a predictor for fatigue after chemoradiotherapy. Female spouses with higher baseline levels of fatigue and burden and caring for patients with lower levels of HRQoL seem risk factors for burden after chemoradiotherapy.

**Conclusions:**

Attention should be paid to caregivers’ burden and fatigue before starting patients’ intense treatment with chemoradiotherapy, as both burden and fatigue before starting treatment may contribute to burden and fatigue after chemoradiotherapy.

## Introduction

Every year, globally, 650,000 patients are diagnosed with head and neck cancer. The most common type of head and neck cancer is head and neck squamous cell carcinoma (HNSCC) which is associated with tobacco and alcohol abuse. Furthermore, in oropharyngeal cancer, part of the tumors are caused by the human papillomavirus (HPV). Patients with HPV-positive tumors have a favorable prognosis when compared to patients with HPV-negative tumors [1]. Standard treatment in patients with locally advanced head and neck cancer (LAHNC) is concomitant chemoradiotherapy with curative intent. This is an intensive treatment accompanied with (visible) consequences and side effects such as severe fatigue, mucositis, and dermatitis [2], which negatively influence the patients’ quality of life and may cause psychological distress [2–4]. Patients with head and neck cancer differ from other cancer patients, as the prevalence of major depressive disorder is the highest among head and neck cancer patients [5].

Considering the aforementioned aspects, social support from patients’ informal caregivers is indispensible during and after treatment. Badr and colleagues found that patients with head and neck cancer and their caregiver cope with distress as a couple [6]. Consequently, caregivers can be burdened by care for their significant other. Lazarus and Folkman introduced the stress theory and described the construct of “appraisal” [7]. This is a cognitive process of an individual to balance environmental demands or stressors in relation to their personal life. This theory and the construct of appraisal is often used in caregiver literature, as burden can be an outcome of this appraisal [8, 9]. Caregiver burden is described by Zarit and colleagues as the extent to which giving care to a significant other is perceived with an adverse effect on their emotional, social, financial, physical, and spiritual functioning [10]. Adelman and colleagues describe that this definition emphasizes the multidimensional toll caregiving may demand on care providers and that giving care to a significant other is a highly individualized experience [11]. Psychopathology may arise when there is an imbalance between demands and caregivers’ personal life [8]. High levels of caregiver burden may remain high after ending treatment, which is shown in a study with caregivers of patients treated for stages II and III esophageal cancer [12]. Studies performed among caregivers of patients with head and neck cancer show that head and neck cancer caregivers have poorer mental health, with higher distress levels when compared to the general population and compared to head and neck cancer patients themselves [13, 14]. Risk factors for poorer mental health and burden are being female, providing more hours of care, having disrupted social interaction, and having disrupted self-care and an increased need for patients’ support [15]. Additionally, fatigue is a frequently mentioned physical symptom of burden and is likely to coincide with burden, which is sparsely studied among caregivers of patients with cancer [16], and not yet studied in this group of caregivers.

This prospective, observational pilot study had two exploratory aims. First, we wanted to explore the course of caregivers’ burden and fatigue in relation to patients’ fatigue, distress, and HRQoL before and after chemoradiotherapy. An important number of caregivers of head and neck cancer patients’ report needing help themselves [17]. Therefore, we aim to identify caregivers with a high level of burden and fatigue and we want to investigate when the levels of burden and fatigue are at their highest. This is in order to identify when support seems to be needed most. Second, we aimed to explore risk factors for developing higher levels of burden and fatigue of caregivers after patients’ treatment with chemoradiotherapy. Based on clinical observations of informal caregivers of patients treated for LAHNC and based on known risk factors for care-related problems supporting a patient with cancer, we think that female gender [18], younger age [18, 19] and being in a spousal relationship with the patient [19–21], caregivers’ higher baseline role problems [22], and caregivers of patients with worse HRQoL [9, 23, 24] have a higher risk for higher levels of burden and fatigue after ending treatment for LAHNC.

## Materials and methods

### Setting and participants

This prospective, observational pilot study was conducted between 2011 and 2013 at the Radboud University Medical Center in the Netherlands. Eligible for participation were patients older than 18 years with LAHNC and who were scheduled for treatment with chemoradiotherapy with curative intent (stages III, IVa, IVb) and their informal caregivers. Patients and caregivers had to be able to give informed consent and read and write in Dutch. Patients receiving the chemoradiotherapy as primary treatment were treated with concomitant chemoradiotherapy during 5.5 weeks. They received accelerated radiotherapy. Patients who were treated with concomitant chemoradiotherapy as postoperative therapy were treated with a conventional chemoradiotherapy schedule and received treatment for 7 weeks. In oropharyngeal cancer patients, HPV positivity was determined by the use of immunohistochemical determination of p16. In case p16 was positive, PCR for HPV was performed. If also the PCR was positive, we identified the patient as HPV positive.

### Procedure

The local medical ethical committee gave permission for the study. The attending oncologist (CH) and/or nurse practitioner (CO) informed the couples during their first visit to the outpatient clinic. If both patient and caregiver gave their informed consent, they were included. The attending oncologist or nurse practitioner extracted the treatment characteristics from the patient’s medical record, including HPV status, postoperative chemoradiotherapy (yes/no), and duration of treatment (weeks). Participants were asked to complete self-report questionnaires at three time points: (T0) prior to start chemoradiotherapy, (T1) 1 week after ending chemoradiotherapy, and (T2) 3 months after the end of chemoradiotherapy. Completing the paper and pencil questionnaire took between 45 and 60 min.

### Questionnaires

A general questionnaire assessed caregivers’ and patients’ demographic characteristics, including gender, age, nationality, education, and employment.

#### Caregiver burden

Caregivers’ burden was assessed by the Self-Perceived Pressure from Informal Care questionnaire (SPPIC) which is a Dutch, validated questionnaire [8]. It measures how personal interests (i.e., possibility to have own thoughts, activities, and/or other roles they want to fulfill in life) interfere with the pressure they perceive as a consequence of giving care to a significant other. Examples of the questions are “As a consequence of the situation of my significant other, less time is available managing my personal life” and “Combining the responsibility for my significant other and my family and work is challenging.” It consists of nine items and is scored on a 5-point Rasch scale. According to the manual of the questionnaire, the scores are dichotomized to 0 (“no!” and “no”) and 1 (“yes!”, “yes,” and “more or less”), and scores range from 0 to 9. Higher scores indicate higher levels of caregivers’ burden. Total scores on the scale were defined as low (0–3), moderate (4–6), and high levels of burden (7–9) [12]. The internal consistency of the questionnaire in this sample was sufficient (*α* = .74).

#### Patient psychological distress and quality of life

Patients completed the Hospital Anxiety and Depression Scale (HADS) [25], which is a 14-item self-assessment questionnaire to assess psychological distress. Each item is rated on a scale from 0 (not at all) to 3 (very much). Total scores range between 0 and 42, with higher scores indicating higher distress. A cutoff score of 11 was used for detecting manifest distress [26]. The scale has been translated and validated in the Dutch general population and showed a good internal consistency (*α* = 0.82–0.90) [27].

The EORTC QLQ-C30 (version 3.0) is a well-validated questionnaire to assess HRQoL. [28] It consists of five functional scales (physical, role, emotional, cognitive, and social functioning), three symptom scales (fatigue, pain, nausea), six single-item scales (dyspnea, sleep disturbance, appetite loss, constipation, diarrhea, financial impact), and the global HRQoL scale. Final scores range between 0 and 100. Higher scores of the functional scales representing better functioning and higher levels of the symptoms scales are representing more symptoms. The internal consistency of this questionnaire is good (*α* = 0.84) [28]. We decided to only use the global HRQoL scale in our exploratory analyses, as this variable gives a more overall view of the well-being while treated with chemoradiotherapy. An increase or decrease of 10 points or more on the subscale HRQoL is regarded as a clinically relevant change [29]. Additionally, the EORTC QoLQ-H&N35 (head and neck module) assesses treatment-related symptoms: six symptom scales (pain, swallowing, senses, speech, social eating, social contact, sexuality), six symptom items (problems with teeth, problems with opening mouth, sticky saliva, coughing, feeling ill), and five additional items (pain medication use, nutritional supplement use, feeding tube, changes in body weight). Higher scores indicate more symptoms. The internal consistency of the subscales is good (*α* = 0.72–0.95) [28].

#### Caregiver and patient fatigue

For caregivers and patients, the severity of fatigue was assessed by the validated subscale fatigue severity of the Checklist Individual Strength (CIS). The 8-item subscale is scored on a 7-point Likert scale. The final score ranges from 8 to 56. Higher scores indicating more fatigue [30]. A cutoff score of 35 and higher was used to indicate severe fatigue. This cutoff score was validated in healthy subjects in the general population and patients with chronic fatigue [31]. The cutoff was used in different studies with patients in different phases of treatment for cancer and caregivers and shows that the scale is sensitive to change in levels of fatigue over time [32–34]. The internal consistency of the subscale is good (*α* = 0.88) [30].

### Statistical analyses

Analyses were performed using Statistical Package for the Social Sciences software version 20 (SPSS Inc. Chicago, IL, USA). When the data of the patient and/or the caregiver were incomplete at any time point, the dyad was excluded for analyses. For sample characteristics, descriptive statistics were used. For continuous variables, Student’s *t* tests (equal distribution expected between groups) or the Mann-Whitney *U* test (equal distribution unlikely) were used. For categorical variables, chi square tests or Fisher’s exact test were performed. Characteristics of dyads who dropped out during the study were compared with the dyads who completed all the measurements. This was performed for age, gender, marital status, level of education, patients’ and caregivers’ baseline fatigue, caregivers’ baseline burden, and patients’ HRQoL and distress.

**Fig. 1:**
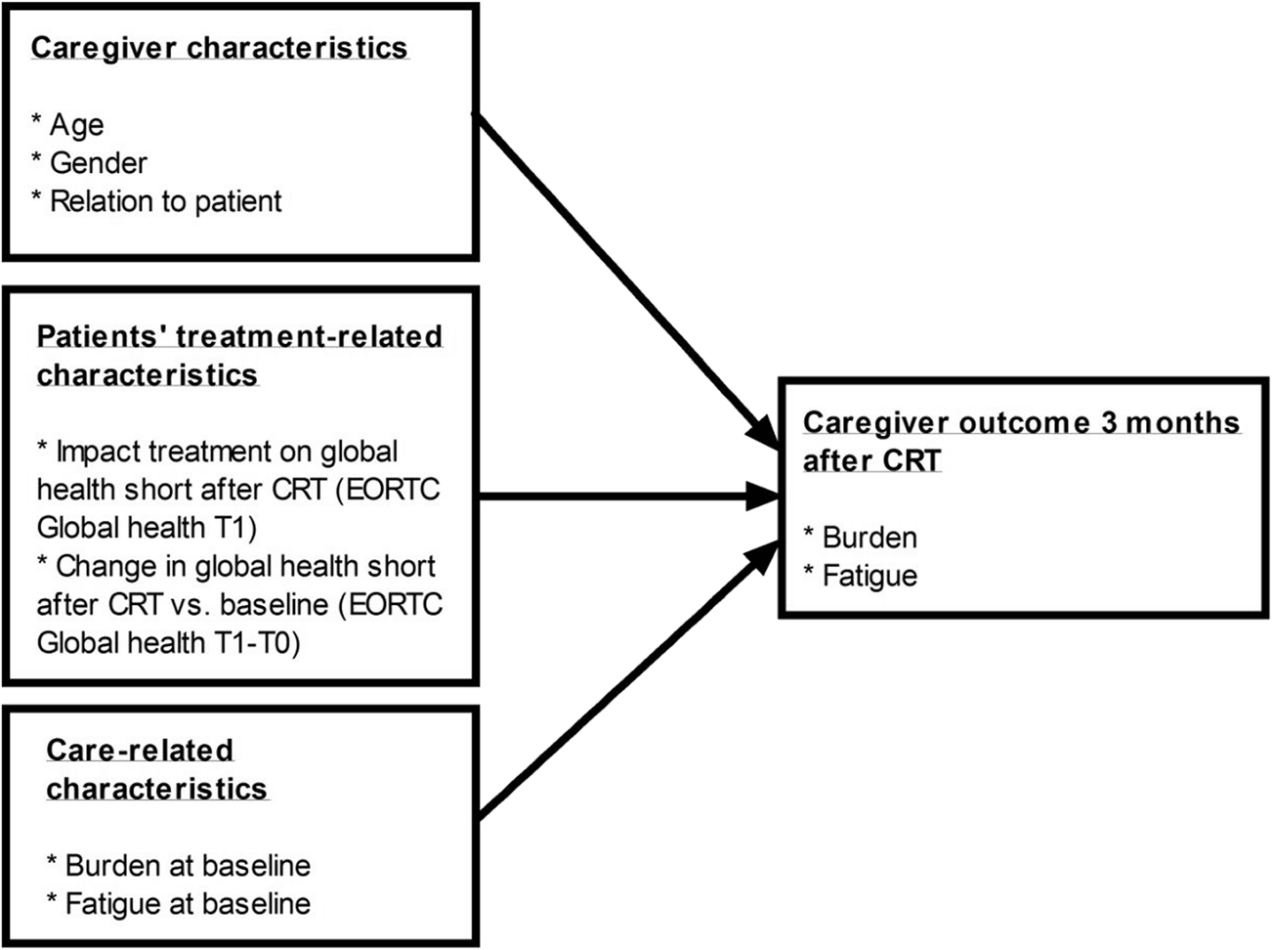
Research model for predicting factors contributing to burden and fatigue of informal caregivers after patients’ chemoradiotherapy

For our first exploratory aim, general linear model repeated measure analysis was performed to determine caregivers’ course of burden, using mean SPPIC scores on all measurement time points (T0, T1, and T2). For determining caregivers’ and patients’ course of fatigue, mean fatigue severity scores on all measurement time points (T0, T1, and T2) were used. Additionally, the same analyses were performed to determine caregivers’ fatigue in relation to patients’ fatigue, using mean scores on all the measurement time points (T0, T1, and T2). To explore the association between patients’ distress and caregivers’ burden and distress, Pearson correlations were performed. For our second exploratory aim, we designed an exploratory model, which is shown in Fig. 1. Linear regression analyses (enter method) were conducted based on the exploratory model. Fatigue and burden on T2 were used as dependent variables. Independent variables were age, gender, relation to the patient, patients’ HRQoL on T1, patients’ difference in HRQoL between T1 and T0, caregivers’ baseline burden, and caregivers’ baseline fatigue. The adjusted *R*^2^ and Beta weights were used for interpretation of the model. The assumptions of linearity, constant error variance, and normality were determined by performing residual analysis. Statistical significance was determined based on a two-sided alpha of 0.05.

## Results

### Caregivers’ and patients’ baseline characteristics

In total, 68 patients were eligible. Eight dyads decided not to participate. Reasons not to participate were that participating was perceived too burdensome (*n* = 4); patient had caregivers, but no principal one (*n* = 2); dyad was not recruited by accident (*n* = 1); or reasons were unclear (*n* = 1). After inclusion, three dyads withdrew from participation for unclear reasons and one patient passed away. The mean recruitment rate was three dyads every month. For analyses, complete data of 56 dyads was available for T0, 49 dyads for T1 (withdrew *n* = 3; incomplete data *n* = 1; patient deceased *n* = 1, recurrent disease *n* = 1, reason unclear *n* = 1), and 45 dyads for T2 (recurrent disease *n* = 2, logistic mistake (*n* = 1), incomplete data *n* = 1). Baseline characteristics are given in Table 1. For age, gender, marital status, level of education, and baseline fatigue, no significant differences were found between caregivers who dropped out (*n* = 11) and those who completed all assessments at all time points (*n* = 45), except for baseline burden, which was significantly lower in the group who dropped out (mean rank 16.6) compared to the group who completed all measurements (mean rank 31.4; *p* = 0.006). Patients who dropped out (*n* = 11) showed no significant differences compared to the patients who completed all measurements (*n* = 45), except for baseline HRQoL, which was significantly higher in the group of patients who dropped out (mean rank 37.6) compared to the patients who completed all measurements (mean rank 25.5; *p* = 0.03). At T0, patients’ health-related functioning was significantly better, and disease-related symptoms were significantly less when compared with data of the EORTC manual reference values (stages III/IV head and neck cancer) [35]. Details are outlined in Table 2.

**Table 1:** Sample characteristics

Characteristics		Caregiver *n* (%)	Patient *n* (%)
Participants		56	56
Gender	Female, *n* (%)	42 (75)	19 (34)
	Male, *n* (%)	14 (25)	37 (66)
Median age, years (ICR)		58 (44–64)	58 (53–63)
Education level (ISCED)	Lower education ≤ 4, *n* (%)	44 (80)	43 (78)
	Higher education > 4, *n* (%)	11 (20)	12 (22)
Relation to patient	Partner, n (%)	39 (70)	–
	Parent, *n* (%)	5 (9)	–
	Child, *n* (%)	7 (13)	–
	Friend, *n* (%)	3 (5)	–
	Other, *n* (%)	2 (4)	–
Employment status	Employed (paid), *n* (%)	32 (58)	26 (47)
	Unemployed, *n* (%)	0 (0)	7 (13)
	Housekeeper, *n* (%)	12 (22)	8 (14.5)
	Disablement insurance act, *n* (%)	2 (4)	6 (11)
	Retired, *n* (%)	9 (16)	8 (14.5)
Duration of treatment	Weeks, median (IQR)	–	5.5 (5–6)
Chemoradiotherapy (CRT)	Postoperative CRT (%)	–	42.9
	Primary CRT (%)	–	57.1
HPV status	Positive, *n* (%)	–	5 (8.9)

*ISCED* International Standard Classification of Education 2011; ≤ 4, secondary, non-tertiary education level; > 4, tertiary education level (bachelor, master, or doctoral level), *HPV* human papilloma virus

**Table 2:** EORTC mean scores of sample, compared to reference data [27]

	Reference data (*n* = 1722); head and neck cancer stage III–IV, mean (SD)	Baseline scores patients (*n* = 56)	*p* value
QLQ-C30			
Global health-related QoL	63.1 (22.4)	73.3 (19.7)	< 0.001^a^
Physical functioning	81.2 (20.2)	87.0 (14.0)	0.004
Role functioning	78.8 (27.9)	72.2 (29.8)	0.111
Emotional functioning	71.2 (24.1)	78.2 (17.5)	0.005
Cognitive functioning	86.4 (19.1)	89.8 (19.3)	0.198
Social functioning	82.2 (24.7)	77.8 (25.5)	0.208
Fatigue	27.6 (25)	21.8 (20.2)	0.04
Nausea and vomiting	5.2 (13.3)	1.9 (7.0)	0.001
Pain	24.9 (26.3)	21.6 (26.4)	0.364
Dyspnea	18.0 (26.6)	6.9 (16.5)	< 0.001^a^
Insomnia	28.5 (32.4)	26.5 (29.2)	0.625
Appetite loss	19.4 (29.3)	8.6 (20.7)	< 0.001^a^
Constipation	11.7 (23.2)	8.0 (18.2)	0.145
Diarrhea	6.1 (16.7)	4.3 (13.0)	0.32
Financial difficulties	18.8 (30.2)	13.2 (13.0)	0.106
QLQ-HN-35			
Pain	29.9 (25.1)	21.3 (19.1)	0.002
Swallowing	27.5 (26.1)	17.8 (23.8)	0.005
Senses	20.0 (30.0)	9.8 (17.1)	< 0.001^a^
Speech	27.1 (27.2)	16.9 (19.7)	< 0.001^a^
Social eating	23.9 (26.7)	13.0 (14.4)	< 0.001^a^
Social contact	13.2 (19.1)	7.7 (13.0)	0.004
Sexuality	32.3 (36.1)	28.1 (21.8)	0.31
Teeth	27.8 (35.0)	10.2 (21.7)	< 0.001^a^
Opening mouth	22.4 (31.9)	30.1 (33.2)	0.099
Dry mouth	31.1 (34.2)	19.2 (23.2)	0.001^a^
Sticky saliva	32.4 (35.4)	20.9 (24.0)	0.001^a^
Coughing	34.9 (32.1)	17.7 (26.1)	< 0.001^a^
Felt ill	21.7 (29.2)	10.3 (19.3)	< 0.001^a^
Pain killers	52.8 (49.9)	61.5 (49.1)	0.205
Nutritional supplements	27.0 (44.4)	28.9 (45.7)	0.772
Feeding tube	18.3 (38.7)	7.6 (26.7)	0.005^a^
Weight loss	41.3 (49.2)	34.6 (48.0)	0.32
Weight gain	25.9 (43.8)	36.5 (48.6)	0.121

^a^Statistically significant difference

### Caregivers’ course of burden and fatigue and patients’ course of fatigue, distress, and HRQoL

Caregivers’ mean scores on burden and the distribution of the level of burden are given in Table 3. Caregivers’ mean scores of burden changed significantly over time (*p* = 0.006) and showed a peak at T1. At T2 after chemoradiotherapy, burden was significantly lower when compared to burden at T0 and T1 (*p* = 0.024 and *p* = 0.001, respectively).

**Table 3:** Mean scores for caregivers’ burden and fatigue and distribution on individual level (> cutoff)

Time point (*n*)	T0 (*n* = 56)	T1 (*n* = 47)	T2 (*n* = 45)
Mean scores (SD)			
Fatigue	24.3 (13)	27.5 (12.4)	22.4 (11.8)
Burden	4.1 (2.4)	4.6 (2.4)	3.2 (2.4)
Score > cutoff *n* (%)			
Fatigue	10 (19)	14 (30)	9 (21)
Burden			
Low	30 (53.6)	20 (42.6)	30 (66.7)
Moderate	20 (35.7)	17 (63.2)	10 (22.2)
High	6 (10.7)	10 (21.3)	5 (11.1)

Caregivers’ mean scores on fatigue and the proportion of severely fatigued caregivers are shown in Table 3. Caregivers’ decrease from T1 to T2 was significant (*p* = 0.029).

There was no statistically significant difference for caregiver found between groups for HPV positivity on all three time points for burden (T0: mean rank 40.6 versus 27.3, *p* = 0.079; T1: mean rank 27.2 versus 23.6, *p* = 0.578; T2: mean rank 32.2 versus 21.9, *p* = 0.093) and fatigue (T0: mean rank 35.3 versus 26.7, *p* = 0.243; T1: mean rank 26.2 versus 23.7, *p* = 0.704; T2: mean rank 29.7 versus 20.9, *p* = 0.144).

Patients’ mean scores on fatigue, distress, and global health are shown in Table 4. Patients’ fatigue increased significantly from T0 to T1 (*p* < 0.001) and decreased significantly from T1 to T2 (*p* < 0.001). Patients’ levels of fatigue were significantly higher at T2 compared to T0 (*p* = 0.026). Patients’ distress changed over time (*p* = 0.012) and showed a peak at T1. Distress increased between T0 and T1 (*p* = 0.02), and decreased again between T1 and T2 (*p* = 0.03), down to baseline levels. There was no difference in distress at T0 and T2. Patients’ HRQoL decreased from T0 to T1 (*p* < 0.001) and recovered from T1 to T2 (*p* < 0.001), up to baseline levels. Moreover, a clinically relevant decrease in HRQoL was observed from T0 to T1 (> 10 points; from 73.3 to 54.3; range 0–100), and this was restored from T1 to T2 (> 10 points; from 54.3 to 72.0; range 0–100).

**Table 4:** Mean scores of patients’ fatigue, distress, and HRQoL and proportion of patients’ fatigue severity and distress

Time point (*n*)	T0 (*n* = 56)	T1 (*n* = 47)	T2 (*n* = 45)
Mean scores (SD)			
Fatigue	23.7 (12.1)	37.8 (12.7)	28.1 (12.7)
Distress	10.4 (7.3)	13.2 (9.0)	10.7 (6.8)
Global health	73.3 (19.7)	54.3 (25.0)	72.0 (15.7)
Score > cutoff (*n*%)			
Fatigue	9 (16)	31 (65)	14 (33)
Distress	25 (46)	26 (54)	25 (56)

Comparing the course of caregivers’ fatigue to patients’ fatigue, patients were significantly more fatigued than their caregivers (*F* = 5.245 (1.81); *p* = 0.025). Fatigue peaked for both patients and caregivers at T1 (*F* = 39,153 (1.81); *p* < 0.001). Patients’ fatigue was lower at baseline but increased significantly faster (*F* = 9.233 (1.81); *p* = 0.003).

### Correlations

At baseline, caregivers’ burden was significantly correlated to patients’ fatigue, distress, and HRQoL (*r* = .28, *p* = 0.04 and *r* = .32, *p* = 0.02 and *r* = − .331, *p* = 0.02, respectively). At T1, caregivers’ burden was significantly correlated to patients’ distress and HRQoL (*r* = .47, *p* = 0.001 and *r* = − .44, *p* = 0.002). At T2, burden was significantly correlated to patients’ distress and HRQoL at T2 (*r* = .411, *p* = 0.005 and *r* = − .413, *p* = .005).

At baseline, caregivers’ fatigue was significantly correlated to patients fatigue at T0 (*r* = .31, *p* = 0.03). Patients’ fatigue at T0 was significantly correlated to distress and HRQoL at T0 (*r* = .452, *p* = 0.001 and *r* = − .657, *p* < 0.001, respectively), T1 (*r* = .623, *p* = <0.001 and *r* = − .723, *p* < 0.001, respectively), and T2 (*r* = .314, *p* = 0.04 and *r* = − .616, *p* < 0.001, respectively).

Patients’ distress was significantly correlated to their HRQoL at T0, T1, and T2 (*r* = .− 564, *p* < 0.001, *r* = − .725, *p* < 0.001 and *r* = − .508, *p* < 0.001, respectively). Caregivers’ fatigue on T0, T1, and T2 was significantly correlated at all time points (*r* = .43, *p* = 0.001, *r* = .51, *p* < 0.001 and .54, *p* < 0.001, respectively).

### Exploration of risk factors for burden and fatigue after chemoradiotherapy

Table 5 shows the results of the regression analysis. Twenty-seven percent of the variance in scores for burden on T2 could be explained by the independent variables. There was no significant attribution of one individual independent variable. However, all seven variables together, i.e., younger age, spousal and female caregivers, higher levels of fatigue and burden on baseline within caregiver, and greater difference in decline in HRQoL within the patient, were found to contribute significantly (*p* = 0.012). Burden and fatigue at baseline contributed most to burden 3 months after chemoradiotherapy (13% of the 27%), followed by gender, patients’ global HRQoL at T1, and relation to the patient. Age and the change in global HRQoL between T1 and T0 contributed less.

**Table 5:** Determinants of burden and fatigue among caregivers of patients with LAHNC, 3 months after completing chemoradiotherapy; final *ß* weight and adjusted *R*^2^ (*n* = 41)

	Burden	Fatigue
Caregiver characteristics		
Gender (male = 0; female = 1)	0.234	0.067
Age	0.059	0.065
Relation to patient (non-spouse = 0; spouse = 1)	0.218	− 0.023
Adjusted *R*^2^	0.08	0.01
Patient treatment-related characteristics		
EORTC GH T1	− 0.222	0.023
EORTC GH T1-T0	0.058	0.25
**∆** Adjusted *R*^2^	0.06	0.04
Caregiver care-related characteristics		
Baseline burden	0.267	− 0.009
Baseline fatigue	0.262	0.609^*^
∆ Adjusted *R*^2^	0.13	0.29
Total *R*^2^	27%	34%

^*^p < 0.01

Thirty-four percent of the variance in fatigue scores was explained by the independent variables. Caregivers’ fatigue at baseline contributed significantly to caregivers’ fatigue on T2 (*p* < 0.01). Caregivers’ care-related characteristics (i.e., burden and fatigue) at baseline contributed most to the explained variance of fatigue scores 3 months after chemoradiotherapy (29% of 34%).

## Discussion

This prospective, observational pilot study of patients with LAHNC and their caregivers is one of the few studies to focus on the course of burden and fatigue of caregivers in relation to patients fatigue, distress, and global HRQoL. It contributes to the existing knowledge about risk factors for burden and fatigue in caregivers of patients short after patients’ end of treatment with chemoradiotherapy.

This study adds valuable knowledge for the identification of caregivers at risk for burden when patients have finished chemoradiotherapy. Female spouses with higher baseline levels of burden and fatigue, and caring for patients with lower levels of global HRQoL seem at higher risk for burden after the end of the intensive treatment of chemoradiotherapy. Adelman and colleagues describe risk factors for caregiver burden, which resemble our findings, such as female sex and cohabitation with the care recipient [11]. They describe caregivers who suffer from sleep deprivation are at higher risk, which seems to be in line with our finding that caregivers’ baseline fatigue contribute to higher levels of burden after end of chemoradiotherapy. Longacre and colleagues describe in a review the psychological health of caregivers of patients with head and neck cancer [15]. They do not find a consistency on the caregivers’ risk on higher levels of burden in relation to the female gender. The need for psychological help, however, is higher among women. Furthermore, they found a relation between treatment-related factors and caregiver burden. No association was found between caregiver distress and patient type of treatment and their functional impairment. They conclude that often no consistency is found in factors contributing to poorer mental health among caregivers, which could be a consequence of different methodology (small sample sizes and lack of longitudinal study design) and terminology (the definition of caregivers, for example).

This study adds valuable knowledge to the literature about the course of caregiver burden. The available data of caregivers’ course of burden during and after treatment is limited and mostly regarding caregivers’ burden during treatment. Nightingale and colleagues reported an increase in burden during radio- and/or chemotherapy, which remained high up to the end of treatment [36]. Badr and colleagues reported constant levels of burden, up to 6 weeks after initiating treatment (radiotherapy alone, and/or in combination with prior surgery and/or chemotherapy) [6]. A recent pilot study of Nightingale and colleagues showed that caregivers of patients receiving radiotherapy, reported higher levels of burden during and 1 month after ending therapy [37]. Our study showed an increase in burden 1 week after chemoradiotherapy and a decrease to baseline levels 3 months after chemoradiotherapy. In order to prevent this rise in caregiver burden and the risk for burden after treatment, support for caregivers at risk for burden may be focused at the start of patient’s treatment. Higher levels of burden and fatigue of the caregiver at the start of patients’ treatment seem to contribute most to their burden after chemoradiotherapy. Based on this study, no conclusions can be drawn about determinants of baseline burden and fatigue and what kind of support for caregivers is needed. This should be determined with future research.

A main risk factor for caregivers’ severe fatigue after patients finish chemoradiation is baseline fatigue. Fatigue is considered an important determinant for general health, and levels of fatigue were higher among caregivers when compared to the general working population [38, 39]. Additionally, fatigue severity was in the range of caregivers of patients in the palliative phase [33]. One could imagine that the intensive treatment with chemoradiotherapy, with daily visits to the hospital, and the impact of treatment-related side effects and their specific care demands may play an important role in caregivers’ fatigue. Caregivers have to combine support for the patient with their own personal life. In this study, more than half of the caregivers combine their role as caregiver with work. It is known that being employed while caring for a significant other is challenging and can negatively influence caregivers’ well-being, especially when caring demands flexibility while the caregivers’ work demands otherwise [40]. Caregivers’ fatigue, where a peak was found 1 week after chemoradiotherapy, is different from the constant levels reported by caregivers of patients in the palliative phase [33]. In order to support caregivers of patients with LAHNC coping with their own fatigue, it is important to know what causes fatigue before starting treatment. Our study shows that fatigue at baseline could be associated with patients’ fatigue, distress, and HRQoL. However, it remains unclear whether this causes caregivers’ fatigue and therefore could be a focus for future research.

Although this study adds valuable knowledge, limitations should be considered. The predefined model for these exploratory analyses, based on clinical observations and supported by theory, contained a large number of predictors in a small sample size. Nevertheless, the exploratory model offers a direction for future studies with a larger sample size to identify caregivers at risk for burden and fatigue [41]. Furthermore, we may question the generalizability of our study for other LAHNC patients and their caregivers. Since the sample size is small, it is possible that our findings are too optimistic; the HRQoL of our sample in comparison to the reference group was significantly better at baseline. A possible explanation could be that the reference group represents stages III and IV head and neck cancer patients, whereas our study only included patients with LAHNC without metastases (i.e., stages III, IVa, IVb). On the other hand, it is possible that our findings are more pessimistic; caregivers with lower levels of burden and patients with higher levels of HRQoL dropped out. An explanation for the latter could be that the caregivers with lower levels of burden and patients with higher levels of HRQoL decided to withdraw from the study since they could not identify themselves with the purpose of the study.

In conclusion, burden of caregivers of LAHNC patients receiving chemoradiotherapy is determined by multiple variables and follows patients’ peak in distress, fatigue, and diminished HRQoL. Female, spousal caregivers who are burdened and fatigued at baseline are important to identify before a patient starts treatment in order to prevent burden after patients’ ending of chemoradiotherapy. Where burden seems to be determined by multiple aspects, caregivers’ fatigue after patients’ curative treatment for LAHNC seems predominantly caused by caregivers’ fatigue at baseline. Fatigue is an important problem to identify, since it is an important determinant of a person’s general health and ability to participate in society. Lastly, problems as a consequence of patients’ treatment for LAHNC are likely to influence both the well-being of caregivers and patients. Therefore, it seems justified to involve both patients and caregivers when designing interventions addressing the aforementioned issues.
